# Structure-Activity Relationship of Hydroxycinnamic Acid Derivatives for Cooperating with Carnosic Acid and Calcitriol in Acute Myeloid Leukemia Cells

**DOI:** 10.3390/biomedicines9111517

**Published:** 2021-10-21

**Authors:** Aviram Trachtenberg, Katarzyna Sidoryk, Somaya Alreate, Suchismita Muduli, Andrzej Leś, Marcin Cybulski, Michael Danilenko

**Affiliations:** 1 Department of Clinical Biochemistry and Pharmacology, Faculty of Health Sciences, Ben-Gurion University of the Negev, Beer Sheva 8410501, Israel; aviramtr@post.bgu.ac.il (A.T.); alreates@post.bgu.ac.il (S.A.); muduli@post.bgu.ac.il (S.M.); 2 Team of Chemistry, Department of Pharmacy, Cosmetic Chemicals and Biotechnology, Łukasiewicz Research Network—Industrial Chemistry Institute, 01-793 Warsaw, Poland; sidorykk@gmail.com (K.S.); marcin.cybulski@ichp.pl (M.C.); 3 Faculty of Chemistry, University of Warsaw, 02-093 Warsaw, Poland; ales@tiger.chem.uw.edu.pl

**Keywords:** structure-activity relationship, hydroxycinnamates, carnosic acid, vitamin D, acute myeloid leukemia

## Abstract

Plant phenolic compounds have shown the ability to cooperate with one another at low doses in producing enhanced anticancer effects. This may overcome the limitations (e.g., poor bioavailability and high-dose toxicity) in developing these agents as cancer medicines. We have previously reported that the hydroxycinnamic acid derivative (HCAD) methyl-4-hydroxycinnamate and the phenolic diterpene carnosic acid (CA) can synergistically induce massive calcium-dependent apoptosis in acute myeloid leukemia (AML) at non-cytotoxic concentrations of each agent. Here, we explored the chemical nature of the synergy between HCADs and either CA, in inducing cytotoxicity, or the active metabolite of vitamin D (calcitriol), in enhancing the differentiation of AML cells. This was done by determining the structure–activity relationship of a series of hydroxycinnamic acids and their derivatives (methyl hydroxycinnamates and hydroxybenzylideneacetones) in combination with CA or calcitriol. The HCAD/CA synergy required the following critical structural elements of an HCAD molecule: (a) the para-hydroxyl on the phenolic ring, (b) the carbon C7–C8 double bond, and (c) the methyl-esterified carboxyl. Thus, the only HCADs capable of synergizing with CA were found to be methyl-4-hydroxycinnamate and methyl ferulate, which also most potently enhanced calcitriol-induced cell differentiation. Notably, the C7–C8 double bond was the major requirement for this HCAD/calcitriol cooperation. Our findings may contribute to the rational design of novel synergistically acting AML drugs based on prototype combinations of HCADs with other agents studied here.

## 1. Introduction

The utilization of small-molecule drugs of plant origin (e.g., paclitaxel, vinblastine, topotecan, etoposide, etc.) in the chemotherapy of solid malignancies and lymphoid leukemias illustrates the importance of phytochemicals for clinical oncology [1]. Plant-derived polyphenols, such as curcumin (CUR), epigallocatechin gallate or resveratrol, have been widely investigated for the anti-cancer activity in both laboratory and clinical studies (see [2,3,4,5,6] for recent reviews). Polyphenols exert strong antiproliferative and cytotoxic effects on various malignant cells in culture by targeting multiple cellular pathways [7,8,9,10,11]. However, when applied alone these agents are usually effective at high micromolar concentrations (e.g., [12,13,14]). Furthermore, polyphenols have poor oral bioavailability and biodistribution, thus their pharmacological effects in rodent cancer models are often evident at doses incompatible with human use [7,15]. One approach to lower the pharmacologically effective concentrations of polyphenols and to reduce their possible side effects is to take advantage of the ability of these compounds to cooperate with other antitumor agents and also with one another in producing enhanced anticancer effects, as demonstrated in a number of studies conducted using various in-vitro and in-vivo models (e.g., [16,17,18,19,20,21]). For instance, we and others have shown that the phenolic diterpene carnosic acid (CA) and the polyphenols curcumin CUR and silibinin can strongly synergize with active vitamin D derivatives at low concentrations to exert enhanced cell differentiation and growth arrest in human [22,23,24,25] and murine [26,27] acute myeloid leukemia (AML) cell lines, and leukemic blasts from patients with AML [28,29]. Furthermore, combined treatment with a standardized CA-rich rosemary extract and low-calcemic vitamin D analogs resulted in a strong cooperative antileukemic effect in syngeneic mouse models of AML, without significant toxicity [26,27]. We have also reported that CUR and CA can strongly cooperate at low concentrations of each compound to synergistically induce robust calcium-mediated apoptosis in AML cells, but not in normal hematopoietic cells, and to inhibit disease progression in a xenograft mouse model of AML [30,31].

**Figure 1:**
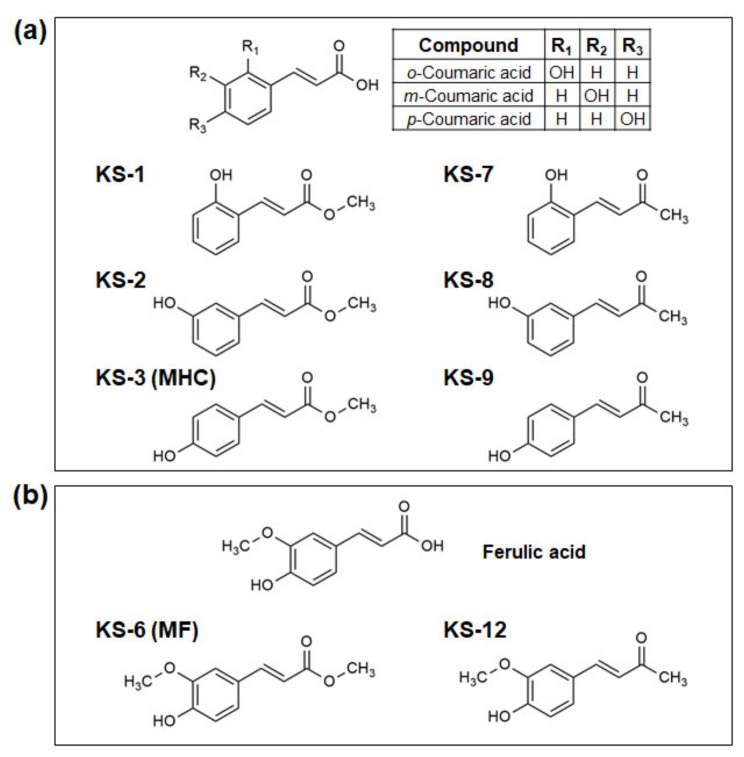
Molecular structures of coumaric and ferulic acids and their derivatives. (**a**) Ortho, meta and para coumaric acids and their derivatives methyl 2-hydroxycinnamate (KS-1), methyl 3-hydroxycinnamate (KS-2), methyl 4-hydroxycinnamate (KS-3), 2-hydroxybenzalacetone (KS-7), 3-hydroxybenzalacetone (KS-8) and 4-hydroxybenzalacetone (KS-9). (**b**) Ferulic acid and its derivatives methyl ferulate (KS-6) and 4-hydroxy-3-methoxybenzalacetone (KS-12).

Hydroxycinnamic acids, e.g., para-coumaric (4-hydroxycinnamic), ferulic and caffeic acids, as well as their derivatives (HCADs) are naturally occurring phenolic compounds synthesized by various plants and mushrooms from the amino acid phenylalanine and are abundant in the diet [32]. The anti-cancer activities of HCADs have been recently reviewed [33,34]. Specifically, several studies demonstrated anti-leukemic effects of caffeic acid phenethyl ester, tetradecyl ester of para-coumaric acid and other HCADs on AML and lymphoid leukemia cells [33,34]. We have found that the HCAD methyl 4-hydroxycinnamate (MHC) is moderately cytotoxic to AML cells but, similar to CUR, can strongly cooperate at low concentrations with CA in inducing calcium-dependent apoptotic cell death. The synergy between MHC and CA was confirmed by combination index analysis [35]. The purpose of the present study was to characterize the structural requirements for MHC-like compounds to effectively cooperate with CA in inducing cytotoxicity and with the hormonal form of vitamin D, 1α,25-dihydroxyvitamin D_3_ (calcitriol), in potentiating cell differentiation. To do so, we investigated the structure–activity relationships of a series of related HCADs (compounds KS-1–KS-12) as well as free para-coumaric and ferulic acid (Figure 1) for their abilities to synergize with CA and calcitriol. The results demonstrated that the para position of the hydroxyl group on the phenolic ring, the C7–C8 double bond and the methyl-esterified carboxyl group, as in MHC (compound KS-3), are critical for the capacity of an HCAD molecule to synergize with CA. Furthermore, we found that the presence of additional groups on the phenolic ring, such as the methoxy group in methyl ferulate (compound KS-6) or hydroxyl groups in compounds KS-4 and KS-5 and methyl caffeate, interferes with this feature of HCADs. On the other hand, the capacity to enhance the prodifferentiation effect of calcitriol appeared to require mainly the existence of the C7–C8 double bond in the HCAD molecules. 

## 2. Materials and Methods

### 2.1. Materials

Carnosic acid (98%) was purchased from Chemlin UK (Nanjing, China). Para coumaric acid (≥98%) was procured from Merck-Sigma-Aldrich (Rehovot, Israel). Ferulic acid (≥98%) and fluo-3/AM were purchased from Santa Cruz Biotechnology (Dallas, TX, USA). Methyl 2-hydroxycinnamate (KS-1), methyl 3-hydroxycinnamate (KS-2), methyl 4-hydroxycinnamate (MHC; KS-3), methyl 2,4-dihydroxycinnamate (KS-4), methyl 2,3,4-trihydroxycinnamate (KS-5)*,* methyl ferulate (KS-6), 2-hydroxybenzylideneacetone (KS-7), 3-hydroxybenzylideneacetone (KS-8), 4-hydroxybenzylidenecetone (KS-9) and 4-hydroxy-3-methoxybenzylideneacetone (KS-12) were synthesized and purified (~96%) by Dr. Katarzyna Sidoryk (Team of Chemistry, Łukasiewicz Research Network—Industrial Chemistry Institute, Warsaw, Poland), as described previously [36]. Methyl 4-hydroxycinnamate (≥98%) and methyl ferulate (≥98%) were purchased from Santa Cruz Biotechnology (Dallas, TX, USA). Methyl caffeate (>98%) was acquired from Cayman Chemical (Ann Arbor, MI, USA). Methyl 4-hydroxybenzoate (MHB, 99%) and methyl 3-(4-hydroxyphenyl)propionate (MHP, 98%) were procured from Alfa Aesar (Haverhill, MA). Methyl 3-hydroxy-4-methoxy-benzoate (MHMB, 98%) and methyl 3-(3-hydroxy-4-methoxyphenyl)propanoate (MHMP, ≥95%) were acquired from Merck-Sigma-Aldrich (Rehovot, Israel) and Activate Scientific (Littleport, UK), respectively. Annexin V-FITC/7-Aminoactinomycin D (7-AAD) Apoptosis Detection Kit (Biogems, Cat# 62700-50, Annexin V FITC Apoptosis Detection Kit) was purchased from BioGems (Chai Wan, Hong Kong, China). Calcitriol (1α,25-dihydroxyvitamin D_3_; >99%) was obtained from Selleck Chemicals (Houston, TX, USA). Mouse anti-Human CD11b (Beckman Coulter, Cat# 6603573, RRID:AB_2890224), Lot# 7012017, FITC conjugated IgM (clone 94), mouse anti-Human CD14 (Beckman Coulter, Cat# 6603262, RRID:AB_2890223), Lot# 7139028, RD1 conjugated, mouse IgG2b (clone 322A-1), mouse IgM, FITC conjugated (Beckman Coulter, Cat# 6603877, RRID:AB_2890227), Lot# 7284017 and, mouse IgG2b, RD1 conjugated (Beckman Coulter, Cat# 6603852, RRID:AB_2890228), Lot# 7301014 were purchased from Beckman Coulter (Fullerton, CA, USA). RPMI 1640 medium and heat-inactivated fetal bovine serum (FBS) were purchased from Gibco-Invitrogen (Carlsbad, CA, USA). Hank’s buffered salt solution (HBSS), Ca^2+^/Mg^2+^-free phosphate buffered saline (PBS), L-glutamine, penicillin, streptomycin, and HEPES were purchased from Biological Industries (Beit Haemek, Israel). The stock solutions of carnosic acid (10 mM) and calcitriol (2.5 µM) were prepared in absolute ethanol. All other test agents were dissolved at 50 mM in DMSO. The precise concentration of calcitriol in ethanol solutions was verified spectrophotometrically at 264 nm (ε = 19,000 M^−1^ cm^−1^). The following solvents were used as vehicle controls: ≤0.1% DMSO or ≤0.1% ethanol for single treatments and ≤0.1% DMSO + ≤0.1% ethanol for combined treatments. 

### 2.2. Cell Culture and Enumeration 

Human KG-1a stem-like leukemia cells (Cat# CCL-246.1, RRID:CVCL_1824), HL60 myoblastic cells (Cat# CCL-240, RRID:CVCL_0002) and U937 myelomonocytic cells (Cat# CRL-1593.2, RRID:CVCL_0007) were obtained from American Type Culture Collection (Rockville, MD). Cells were cultured in RPMI 1640 medium supplemented with 10% FBS, L-glutamine (2 mM), penicillin (100 U/mL), streptomycin (0.1 mg/mL), and 10 µM HEPES (pH = 7.4) in a humidified atmosphere of 95% air and 5% CO_2_, at 37 °C. Routine testing for mycoplasma contamination was carried out once a month using a Myco-Blue Mycoplasma Detector (Vazyme Biotech Co. Ltd., Nanjing, China) according to the manufacturer’s instructions. Briefly, cells were centrifuged at 500× *g* for 5 min in 1 μL of the supernatant, the negative control (PCR-grade water) and positive control were mixed with Myco-Blue Enzyme (1 μL) and MycoBlue Buffer (24 μL), respectively, in separate PCR tubes. Samples were incubated at 60 °C for 1 h and followed by color detection according to the instructions. For experiments, cells were seeded at a concentration of 1.0–2.5 × 10^5^ cells/mL in 24- or 6-well plates, for 8–72 h. Cells were enumerated in Vi-Cell XR cell viability analyzer (Beckman Coulter Vi-Cell XR cell counter (ISO 6 clean room) Vicell, RRID:SCR_019664) version# 2.4 (Beckman Coulter, Fullerton, CA, USA) using an automatic trypan blue exclusion assay, as described previously [35]. The number of viable (trypan blue-impermeable) cells was counted directly. The percent of cell death was calculated as the number of dead (trypan blue-positive) cells relative to the total (viable + dead) cell count. 

### 2.3. Annexin V/7-Aminoactinomycin D Assay

Cells (3 × 10^5^/mL) were washed with PBS and then stained with annexin V-APC and 7-AAD in binding buffer, as described previously [30,35]. Percentages of apoptotic cells were determined by flow cytometry in a Gallios instrument (Beckman Coulter Gallios Flow Cytometer, RRID:SCR_019639; Beckman Coulter, Miami, FL, USA). For each analysis, 10,000 events were recorded, and the data were processed using the Kaluza software (GalliosTM Kaluza, RRID:SCR_016700) version #2.1 (Beckman Coulter, Miami, FL, USA). Annexin V-positive/7-AAD-negative cells were considered to be early apoptotic and cells positive for both annexin V and 7-AAD to be late-apoptotic. 

### 2.4. Determination of Cell Differentiation

Cells were seeded at 5 × 10^4^ cells/mL and treated with test agents or vehicle (≤0.2% ethanol) for 96 h. Aliquots of 5 × 10^5^ cells were harvested, washed with PBS, and incubated for 45 min at room temperature with 0.3 μL anti-human CD11b-FITC and 0.3 μL anti-human CD14-RD1 to determine the expression of myeloid surface antigens CD11b and CD14, respectively, by flow cytometry as described previously [37,38]. For each analysis 10,000 events were recorded, and the data were analyzed using the Kaluza version #2.1 software (Beckman Coulter, Miami, FL, USA). 

### 2.5. Cytosolic Calcium Assay 

Steady state cytosolic Ca^2+^ levels were determined as described previously [30,35]. Briefly, cells (5 × 10^5^/mL) were preloaded with the calcium-sensitive fluorescence indicator Fluo-3/AM (2.5 μM) in CaCl_2_ (2 mM)-supplemented Ringer’s solution, for 30 min at room temperature in the dark. Cells were then washed with Ca^2+−^ free Ringer’s solution and resuspended in the same buffer. Flow cytometric measurement of Fluo-3 fluorescence was performed within 1 min of adding test compounds to the cell suspension. The data are expressed as the geometric mean Fluo-3 fluorescence intensity (MFI). 

### 2.6. Western Blot Analysis

Western blotting was performed using whole cell extracts, as described previously [35]. Briefly, cells were lysed in ice-cold lysis buffer containing 50 mM, HEPES (pH 7.5), 150 mM NaCl, 10% (*v/v*) glycerol, 1% (*v/v*) Triton X-100, 1.5 mM EGTA, 2 mM sodium orthovanadate, 20 mM sodium pyrophosphate, 50 mM NaF, 1 mM DTT and 1:50 Complete™ protease-inhibitors cocktail (Roche Molecular Biochemicals, Mannheim, Germany) and centrifuged at 20,000× *g*, 10 min, 4 °C. Supernatant samples (10–30 μg protein) were subjected to SDS-PAGE and then electroblotted into a nitrocellulose membrane (Whatman, Dassel, Germany). The membranes were blocked with 5% milk for 1 h and incubated with primary antibodies overnight at 4 °C followed by incubation with HRP-conjugated secondary antibodies anti-rabbit (Jackson ImmunoResearch Laboratories, Inc., West Grove, PA, USA) and anti-mouse (GE Healthcare, Pittsburgh, PA, USA) for 1 h. The protein bands were visualized using the WESTAR ECL HRP substrate (CYANAGEN, Bologna, Italy). The following primary antibodies were used: caspase-3 (Santa Cruz Biotechnology Cat# sc-7272, RRID:AB_626803; 1:500), Lot# J219 from Santa Cruz Biotechnology (Santa Cruz, CA, USA); cleaved caspase-3 (Cell Signaling Technology Cat# 9661, RRID:AB_2341188; 1:1000), Lot# 45 from Cell Signaling Technology (Danvers, MA, USA) and poly(ADP-ribose) polymerase (PARP) (Enzo Life Sciences Cat# BML-SA253, RRID:AB_2283566; 1:5000), Lot# 10021421 from Enzo Life Sciences (Farmingdale, NY, USA).

### 2.7. Molecular Modeling of the Formation of CA Anion-HCAD Complexes 

The quantum mechanical density functional theory (DFT) calculations with the B3LYP functional and the 6-31G(d,p) Gaussian basis set with the Grimme’s empirical dispersion correction [39] was used for modeling of the 3D molecular structure of the complexes formed by carnosic acid CA anions and selected HCAD molecules (KS-1, KS-3, KS-7 and KS-9). The optimization of the molecular structures was performed with the Berny’s optimization algorithm. All the calculations were done at the Interdisciplinary Centre for Mathematical and Computational Modelling (ICM, University of Warsaw). The intermolecular interaction energy or the binding energy (BE) was estimated with the following formula:

BE = E(complex) − {E(carnosic acid anion) − E(KS-x)}

where E denotes the DFT energy and x = 1, 3, 7, 9. The binding energy was calculated according to the method usually applied for the calculations of the intermolecular interaction energy with the correction known as the basis set superposition error or the counterpoise correction [40]. Such a correction originates from the fact that a more realistic estimation of the intermolecular interaction energy can be obtained when the energy of each component of the complex is calculated with the use of the atomic basis set of the entire complex.

### 2.8. Statistical Analysis 

All experiments were conducted at least three times. The number of independent experiments (*n*) is indicated in the legends to figures. Statistically significant differences between two experimental groups were estimated by the unpaired two-tailed Student’s *t* test. The significance of the differences between the means of several subgroups was assessed by one-way ANOVA with Tukey’s or Dunnett’s multiple comparison post-hoc analysis. *p* values less than 0.05 were considered statistically significant. The synergy between the effects of two compounds was determined as described previously [24,35]. Briefly, two compounds (A and B) were considered to show enhancement in the particular experiment if the effect of their combination (AB) was larger than the sum of their individual effects (AB > A + B), the data being compared after subtraction of the respective control values from A, B and AB. The statistical analyses were performed using GraphPad Prism 6.0 software (GraphPad Software, San Diego, CA, USA). 

## 3. Results

### 3.1. Essential Structural Features of Hydroxycinnamic Acid Derivatives Required for the Synergy with Carnosic Acid in Inducing Cytotoxicity to AML Cells 

In order to determine the structural requirements of HCADs for the cooperation with CA, we synthesized two series of coumaric and ferulic acid derivatives: methyl hydroxycinnamates and hydroxybenzylideneacetones.

Both series of coumaric acid derivatives included compounds with a hydroxyl group at the ortho (KS-1 and KS-7), meta (KS-2 and KS-8) or para (KS-3/MHC and KS-9) positions of the phenolic ring (Figure 1a). Ferulic acid derivatives were represented by methyl hydroxycinnamate/methyl ferulate (KS-6) and hydroxybenzylideneacetone (KS-12), as shown in Figure 1b. We first tested these HCADs, as well as free para-coumaric acid (pCouA) and ferulic acid (FerA) for the antiproliferative/cytotoxic activity in KG-1a cells. For this purpose, cells were incubated with each compound at 5–25 µM, for 72 h, followed by cell enumeration using the Trypan Blue exclusion assay.

**Figure 2:**
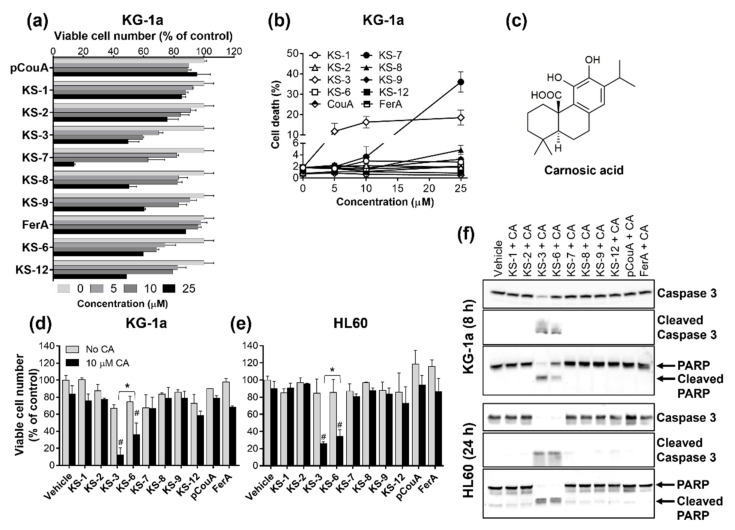
The hydroxycinnamic acid derivatives methyl 4-hydroxycinnamate (KS-3) and methyl ferulate (KS-6), but not their chemical analogs, synergize with carnosic acid in inducing cytotoxicity to AML cells. KG-1a and HL60 cells were treated with 5 µM para-coumaric acid (pCouA), ferulic acid (FerA) or their derivatives, 10 µM carnosic acid (CA), alone or in combination followed by the Trypan Blue exclusion assay (72 h; (**a**,**b**,**d**,**e**)) or Western blot analysis (8 h and 24 h; (**f**)). (**a**,**b**) Effects of the indicated compounds on the number of viable cells (**a**) and the percent of cell death (**b**) in KG-1a cell cultures. (**a**,**b**) The data are the means ± SD (*n* = 3). (**c**) The molecular structure of carnosic acid. (**d**,**e**) Effects of the indicated compounds on the number of viable cells of KG-1a (**d**) and HL60 (**e**) cells. (**d**,**e**) The data are the means ± SD (*n* = 3–6). *, *p* < 0.05, significant difference between the KS-3+CA and KS-6+CA groups. #, *p* < 0.05, effect of a combination vs. sum of the effects of single agents (*n* = 6); Student’s *t* test. (**f**) Caspase-3 and PARP cleavage in KG-1a and HL60 cells following treatment with the indicated combinations of pCouA, FerA or their derivatives, together with CA.

As shown in Figure 2a, all the tested compounds were capable of reducing the number of viable cells in a concentration-dependent manner to a varying extent. KS-7 was found to be the most potent agent, whereas the maximal inhibitory effects of KS-1, pCouA and FerA did not exceed 10–15%. Of all the above compounds, only KS-3 and KS-7 exhibited mild-to-moderate cytotoxicity, as indicated by an increase in the percent of cell death (Figure 2b). Cytotoxicity of the rest of the single agents was ≤5% even at the highest concentration tested (25 µM). 

To explore whether, similar to KS-3/MHC [35], other structurally related HCADs can cooperate with CA (Figure 2c), we counted KG-1a and HL60 cells following the exposure to the synthesized compounds (KS-1–KS-12), pCouA and FerA at their lowest tested concentration (5 µM; Figure 2a,b) and CA at the non-cytotoxic concentration of 10 µM [30,31,35], alone and in combination, for 72 h. Surprisingly, the results demonstrated that, besides KS-3/MHC, only KS-6/MF was able to synergize with CA in reducing the number of viable AML cells (Figure 2d,e). Still, the KS-3+CA combination was found to be significantly more effective than KS-6+CA. Likewise, only these two combinations were capable of inducing apoptosis in KG-1a and HL60 cells, as evidenced by caspase-3 and PARP cleavage (Figure 2f). 

**Figure 3:**
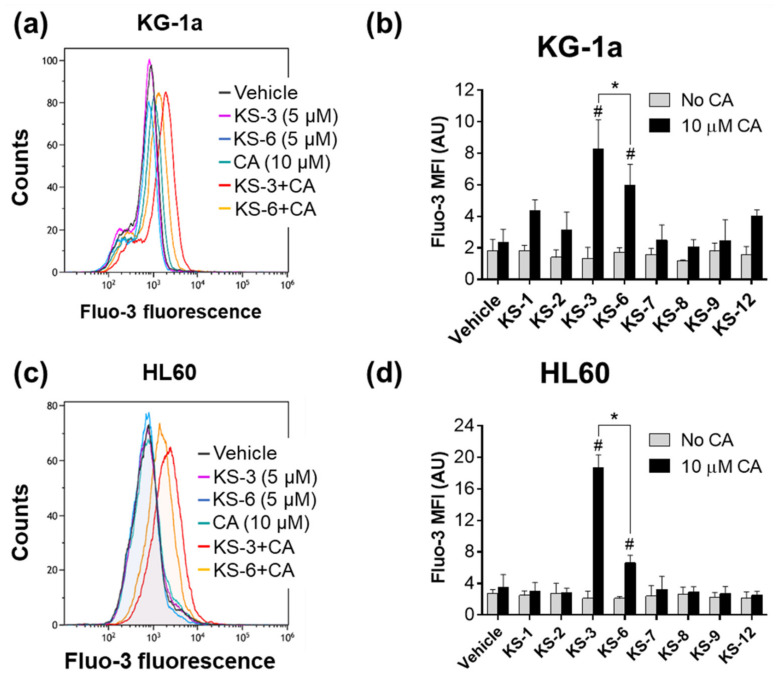
KS-3 and KS-6, but not their chemical analogs, synergize with carnosic acid in inducing a rapid elevation of cytosolic Ca^2+^ levels in AML cells. KG1a and HL60 cells were treated with 5 µM of the indicated hydroxycinnamic acid derivatives, 10 µM carnosic acid (CA) or their combinations for 10 s, followed by determination of Fluo-3 fluorescence by flow cytometry. (**a**,**c**) Typical flow cytometric data of Fluo-3 fluorescence obtained in representative experiments. (**b**,**d**) Averaged Fluo-3 geometric mean fluorescence intensity (MFI) ± SD (*n* = 5). *, *p* < 0.05, significant difference between the KS-3+CA and KS-6+CA groups. #, *p* < 0.05, effect of a combination vs. sum of the effects of single agents; one-way ANOVA with Dunnett’s post-hoc analysis.

We have previously reported that apoptosis induction by both CUR+CA and MHC/KS-3+CA is mediated by intracellular calcium mobilization [30,35]. Here, we confirmed that distinct pro-apoptotic features of the KS-3/CA and KS-6/CA combinations were associated with their significant capacity to induce a rapid elevation of the cytosolic Ca^2+^ levels in both KG-1 (Figure 3a,b) and HL60 (Figure 3c,d) cells, whereas the other tested HCAD/CA combinations produced only a minor or no effect. These experiments again demonstrated that KS-3+CA was more effective, compared with KS-6+CA (Figure 3b,d).

**Figure 4:**
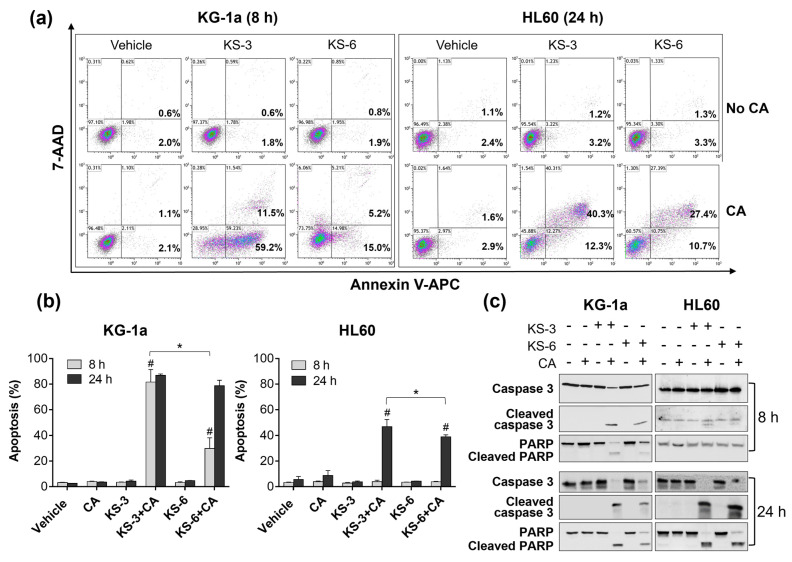
Comparison between the apoptosis-inducing effects of the combinations of KS-3 and KS-6 with carnosic acid. KG-1a and HL60 cells were cultured with 5 µM KS-3 or KS-6, 10 µM carnosic acid (CA) or their combinations, for 8 or 24 h. The extent of apoptosis was determined by the annexin V/7-AAD binding assay or Western blotting. (**a**) Typical flow cytometric data obtained in representative experiments. (**b**) Averaged percentages of apoptotic (early + late) cells as exemplified in *panel* (**a**). The data are the means ± SD (*n* = 5). *, *p* < 0.05, significant differences between the indicated groups; Student’s *t* test. #, *p* < 0.05, effect of a combination vs. sum of the effects of single agents; Student’s *t* test. (**c**) Caspase-3 and PARP cleavage in KG-1a and HL60 cells following treatment with KS-3 or KS-6, alone or together with CA.

Further comparison between the two combinations revealed that KS-3+CA was also more efficient than KS-6+CA in inducing apoptosis, as indicated by higher levels of annexin-V/7-AAD binding (Figure 4a,b), caspase-3 and PARP cleavage (Figure 4c) and blebbing or shrinkage of acridine orange/ethidium bromide-stained nuclei (Suppl. Figure S1) in KS-3+CA-treated cells. These effects were both cell type- and time-dependent. Particularly, in KG-1a cells, marked increases in the above apoptotic markers were evident as early as at 8 h (Figure 4a–c and Suppl. Figure S1a,c) while practically no such increases were observed at this time point in HL60 cells (Figure 4b,c and Suppl. Figure S1d,f).

**Figure 5:**
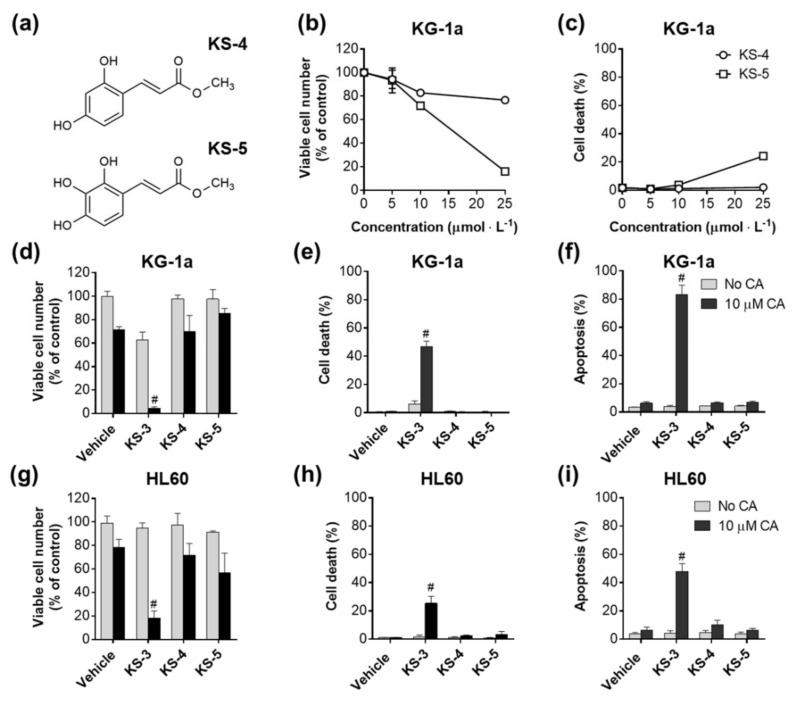
Additional hydroxyl groups on the phenolic ring of KS-3 impair its ability to cooperate with carnosic acid. (**a**) Molecular structures of methyl 3-(2,4-dihydroxyphenyl)acrylate (KS-4) and (E)-methyl 3-(2,3,4-trihydroxyphenyl)acrylate (KS-5). (**b**,**c**) Concentration-dependent effects of KS-4 and KS-5 alone on the number of viable cells (**b**) and the percent of cell death (**c**) in KG-1a cell cultures following 72 h of incubation. (**d**,**e**,**g**,**h**) Effects of KS-3 and its analogs (5 µM) and carnosic acid (CA), alone and in combination, on the number of viable cells (**d**,**g**) and the percent of cell death (**e**,**h**) in KG-1a (**d**,**e**) and HL60 (**g**,**h**) cells (72 h). (**f**,**i**) Effects of the indicated KS-3 and its analogs (5 µM) and CA, alone and in combination, on apoptosis (early+late) induction in KG-1a (**f**) and HL60 (**i**) cells (24 h). The data are the means ± SD (*n* = 3). #, *p* < 0.05, effect of a combination vs. sum of the effects of single agents (*n* = 5); Student’s *t* test.

Collectively, the above data indicate that the para position of a hydroxyl group on the phenolic ring and modification of the carboxyl group to methyl ester, but not methyl ketone, are essential structural requirements allowing coumaric and ferulic HCADs to effectively cooperate with CA in inducing apoptosis in AML cells. Further, these results imply that the presence of other chemical substituents on the KS-3 phenolic ring in addition to the para-OH, e.g., the meta-methoxy group as in KS-6, may interfere with the capability of HCADs to synergize with CA. To test this hypothesis, we examined cytotoxicity of methyl caffeate whose phenolic ring contains meta-hydroxyl besides para-hydroxyl (Suppl. Figure S2a) and newly synthesized KS-3-like compounds in which ortho hydroxyl (KS-4) or both ortho and meta hydroxyls (KS-5) were added (Figure 5a). When applied at 5 25 µM to KG-1a cells for 72 h, the three compounds reduced cell number/viability to varying extents (Suppl. Figure S2b and Figure 5b,c). Yet, in contrast to KS-3, none of these agents was capable of synergizing with CA, as determined by the ATP-based cell viability assay (Suppl. Figure S2c,d), Trypan Blue exclusion assay (Figure 5d,e,g,h) or annexin-V/7-AAD assay (Figure 5f,i) in KG-1a cells (Figure 5d,e,f) and HL60 cells (Figure 5g,h,i). Taken together, the above results support the notion that additional groups on the phenolic ring of KS-3 impair or abolish its ability to cooperate with CA.

**Figure 6:**
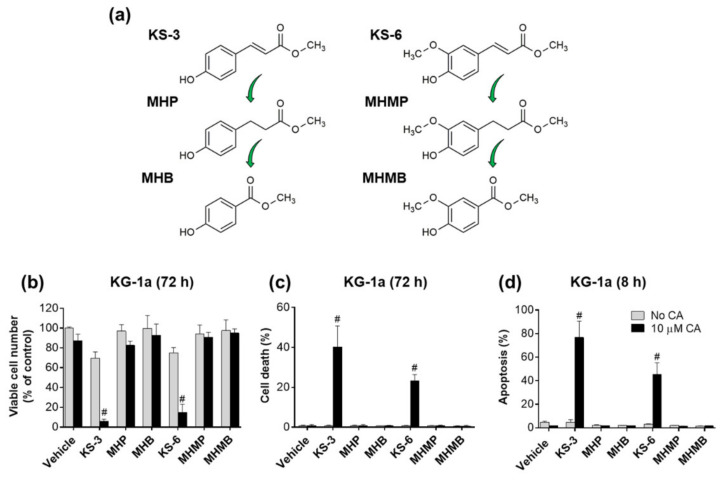
Cooperative activity of KS-3 and KS-6 with CA depends on the C7–C8 carbon-carbon double bond of the α, β unsaturated carbonyl moiety. (**a**) Molecular structures of KS-3, methyl 3-(4-hydroxyphenyl) propionate (MHP), methyl 4-hydroxybenzoate (MHB), KS-6, methyl 3-(3-hydroxy-4-methoxyphenyl) propanoate (MHMP) and methyl 3-hydroxy-4-methoxy-benzoate (MHMB). KG-1a cells were cultured with the indicated phenolic compounds at 5 µM, carnosic acid (CA) at 10 µM, or their combinations, followed by the Trypan Blue exclusion assay (72 h; (**b**,**c**)) or the annexin V/7-AAD binding assay (8 h; (**d**)). (**b**,**c**) Effects of the indicated compounds on the number of viable cells (**b**) and the percent of cell death (**c**) in KG-1a cell cultures. (**d**) The extent of apoptosis (early + late) was determined by flow cytometry, and data are the means ± SD (*n* = 5). #, *p* < 0.05, effect of a combination vs. sum of the effects of single agents; Student’s *t* test.

To examine the role of the α,β-unsaturated carbonyl moieties of KS-3 and KS-6 in this cooperation, we compared the effects of the two HCADs (Figure 6a) and their analogs that have a saturated C7–C8 bond (methyl [3-(4-hydroxyphenyl)] propionate [MHP] and methyl 3-[(3-hydroxy-4-methoxyphenyl)]propanoate [MHMP]) or a truncated side chain (methyl 4-hydroxybenzoate [MHB] and methyl 3-hydroxy-4-methoxybenzoate [MHMB]) (Figure 6a). Remarkably, none of these modified compounds, applied at 5 µM, either alone or together with CA, was able to significantly reduce viable cell numbers (Figure 6b), increase the percent of cell death (Figure 6c) or induce apoptosis (Figure 6d). These results suggest that the C7–C8 double bond of an HCAD is also critical for the cooperation with CA.

### 3.2. Molecular Modeling of the Formation of HCAD ^…^ CA Anion Complexes

Since the above in vitro experiments demonstrated that among all the tested HCADs only KS-3 and, to a lesser extent, KS-6 could synergize with CA, we next explored the potential for a direct intermolecular interaction between KS-3 or KS-6 and the CA anion using in-silico molecular modeling. Three other KS compounds (KS-1, KS-7, and KS-9), which were unable to cooperate with CA in AML cell cultures, were used as negative controls in the modeling studies.

**Figure 7:**
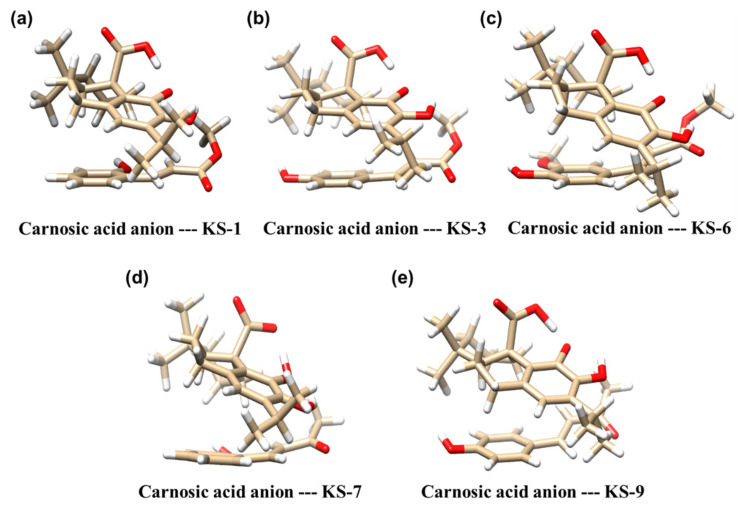
Molecular models of the complexes composed of the carnosic acid anion and selected KS compounds. The aromatic rings of the KS molecules are seen at the bottom of each complex (**a**–**e**). The carnosic acid anion is located above the aromatic ring of KS. The optimal structure of the carnosic anion in the complexes shows the intramolecular hydrogen bond -COO^−^—OH in the form of -COOH—O^−^ as a result of proton transfer between oxygen atoms (see (**a**–**c**,**e**)). The white, gold and red colors correspond to hydrogen, carbon and oxygen atoms, respectively.

The molecular structures of the five putative complexes appeared to have closed forms where both components are positioned almost parallel to each other (Figure 7). Their 3D structures are visible with the use of molecular graphics software (e.g., RasMol, Jmol) based on the Cartesian coordinates available in the Supporting Information (Suppl. Table S1). One can see that no intermolecular hydrogen bonds appear, in contrary to a strong intramolecular H-bond in the CA anion between the COO^−^ and -OH groups. This H-bond is better described as an interaction between COOH and O^−^ resulting from the proton transfer, as it is seen in Figure 7a–e. The geometry of the KS—CA anion molecular complexes is reminiscent of a half-opened oyster. The angle between the aromatic ring of the KS molecule and of the CA anion is about 44°–45° degrees.

As presented in Table 1, the estimated binding energy (BE) of the above complexes is 3–4 times stronger than that of the two water molecules (about 5 kcal/mol). This suggests that, in aqueous solution, these putative complexes can be stable, and, perhaps, are able to reach and be transported into the cells in culture. The KS-3—CA complex exhibited the highest negative BE (Table 1), which correlated with the strongest cooperative cytotoxicity of the KS-3+CA combination, suggesting that this complex might be the most stable among the four analyzed complexes. In contrast, the one containing KS-7, the hydroxybenzylideneacetone counterpart of KS-3 (see Figure 1), forms relatively the weakest bond. It suggests that this complex can dissociate faster than other complexes in the presence of polar molecules, forming the nearest neighbor environment (e.g., the amino acid residues in the active site of the target enzyme or receptor). The negative BE of the KS-6—CA complex (~−16 kcal/mol) was found to be relatively low compared to that of the KS-3—CA complex (~−19 kcal/mol). Therefore, it appears that although KS-6 can cooperate with CA in inducing cytotoxicity, this putative complex is still less stable than KS-3—CA. 

It is worth to note that the KS—CA complexes are bound by stacking interactions, although both components are polar molecules. Another interesting feature of these complexes is the strong interaction between the -COO^−^ anionic group and the OH-group on the CA component. It is likely that there exists some equilibrium, where the proton moves almost freely according to the following scheme:

R-COO^− …^ OH-R ← → R-COOH ^… −^O-R

where R denotes remaining fragments of the CA molecule. Such a unique polar fragment of the CA molecule can interact in a unique way with the surrounding molecules of the environment. 

### 3.3. Hydroxycinnamic Acid Derivatives Can Enhance the Differentiation of AML Cells Induced by Calcitriol

AML is characterized by a differentiation block leading to accumulation of highly proliferative immature myeloid blasts. Therefore, differentiation-based therapy is a promising approach for AML treatment [41]. We have previously shown that (pro-)electrophilic polyphenols and drugs can enhance monocyte/macrophage differentiation of AML cells triggered by active vitamin D derivatives or other agents, without inducing cytotoxicity [24,25,38].

**Figure 8:**
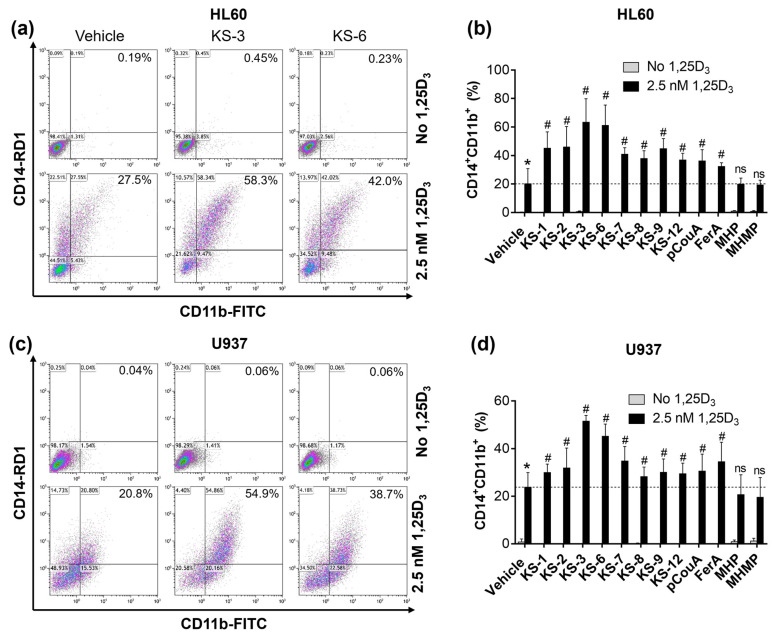
Hydroxycinnamic acids and their derivatives enhance the differentiation-inducing activity of calcitriol in AML cells. HL60 (**a**,**b**) and U937 (**c**,**d**) cells were treated with para-coumaric acid (pCouA), ferulic acid (FerA) or their derivatives (5 µM), calcitriol (1,25D_3_; 2.5 nM) or their combinations, for 96 h. Expression of surface myeloid differentiation markers was measured by flow cytometry. (**a**,**c**) Typical flow cytometric data obtained in representative experiments. The percentages of cells that are double-positive for CD14 and CD11b (CD14^+^CD11b^+^) are shown in the upper right quadrants. (**b**,**d**) Averaged percentages of cells double-positive for CD14 and CD11b. The data are the means ± SD (*n* = 5). *, *p* < 0.05 vs. vehicle. #, *p* < 0.05, effect of a combination vs. sum of the effects of single agents; Student’s *t* test; ns—non-significant.

Here we found that, while having no effect when applied alone for 96 h, most HCADs, as well as pCouA and FerA, could also significantly potentiate, to a varying extent, the pro-differentiation effect of a low concentration of calcitriol (2.5 nM) in HL60 cell (Figure 8a,b) and U937 cells (Figure 8c,d). This is evidenced by significantly higher percentages of cells, double-positive to CD14 (a monocytic surface differentiation marker) and CD11b (a general myeloid marker), observed in combination-treated cells compared with the effect of calcitriol alone (Figure 8b,d). Notably, KS-3 and KS-6 were found to be significantly stronger differentiation enhancers than the other tested compounds (Figure 8b,d and Suppl. Table S2). On the other hand, MHP and MHMP, which lack the double C7–C8 carbon–carbon bond (Figure 6a), had no potentiating effects (Figure 8b,d). These results indicate that the potentiation of calcitriol-induced differentiation by pCouA, FerA and their derivatives is largely dependent on the presence of α, β-unsaturated carbonyl and is stronger when HCADs also have a hydroxyl group at the para position of the phenolic ring and a methyl-esterified carboxyl group. 

**Figure 9:**
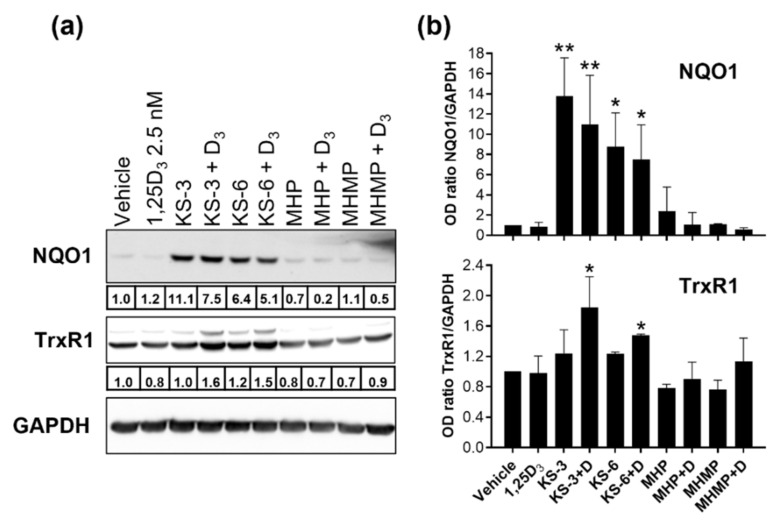
Induction of NQO1 and TrxR1 protein expression in HL60 cells treated with KS-3 or KS-6, but not with MHP or MHMP. Cells were incubated with the indicated phenolic compounds (5 µM), calcitriol (1,25D_3_ or D_3_; 2.5 nM) or their combinations, for 48 h. (**a**) NQO1 and TrxR1 protein expression analyzed by Western blotting. Integrated Density Values (IDVs) of the indicated protein bands normalized to IDVs of respective GAPDH bands are shown below corresponding blot images. Representative blots of three similar experiments are presented. (**b**) Averaged relative protein levels. The data are the means ± SD (*n* = 3). *, *p* < 0.05 and **, *p* < 0.01 vs. vehicle.

We have previously reported that the enhancement of the pro-differentiation activity of vitamin D derivatives by electrophilic compounds containing an α, β-unsaturated carbonyl (e.g., fumaric acid esters) or by CA, which is easily oxidized to an ortho-quinone-type electrophile [42], is attributed to the ability of these electrophiles to activate the transcription factor nuclear factor-erythroid factor 2-related factor 2 (Nrf2) [28,38]. We, thus, compared the effects of KS-3 and KS-6 to those of MHP and MHMP on the protein expression of NAD(P)H:quinone oxidoreductase 1 (NQO1) and thioredoxin reductase 1 (TrxR1), the known Nrf2-responsive gene products [43,44]. As shown in Figure 9, the protein levels of NQO1 and, to a lesser extent, TrxR1 were significantly increased in HL60 cells treated with either KS-3 or KS-6 in the absence or presence calcitriol. In contrast, MHP or MHMP had only a minimal or no effect on the expression of these proteins. 

## 4. Discussion

We have recently reported that, similar to curcumin, methyl 4-hydroxycinnamate (KS-3) can synergistically cooperate with CA at low, non-cytotoxic concentrations of each agent to kill AML cells [35]. In order to explore the chemical nature of this synergy, we characterized here the structure-activity relationship for coumaric and ferulic acids and their methyl ester and methyl ketone derivatives to cooperate with CA in inducing cytotoxicity. We also determined the structural requirements for these compounds to potentiate calcitriol-induced myeloid differentiation of AML cells. 

One of the major novel findings of the present study is that synergistic HCAD/CA cooperation requires the following critical structural features of a HCAD: (a) the para position of the hydroxyl group on the phenolic ring, (b) the C7–C8 double bond and (c) the methyl-esterified carboxyl group. This conclusion is based on the evidence showing that, under otherwise equal conditions, the lack of any one of these features leads to the complete loss of HCAD/CA synergy in reducing viable cell numbers (Figure 2d,e), inducing apoptosis (Figure 2f and Figure 4), and apoptosis-associated raising of cytosolic calcium levels (Figure 3) in AML cells. Interestingly, the presence of other chemical groups on the phenolic ring, in addition to the para hydroxyl group, was found to impair (KS-6) or even abolish (methyl caffeate or compounds KS-4 and KS-5) the capacity of an HCAD to cooperate with CA (Suppl. Figure S2, Figure 5 and Figure 6). Collectively, these data support the notion that methyl 4-hydroxycinnamate (KS-3) is likely to represent an optimal HCAD structure required for effective synergistic cooperation with CA. 

Although the precise roles of the above essential chemical properties of KS-3 in this synergy remain to be fully elucidated, each of these features was shown to be important for the biological effects of different agents. Particularly, phenolic compounds with a hydroxyl group in the para position were found to exhibit stronger biological activities, compared with similar molecules with hydroxyls at other positions. For instance, coumarins with para-hydroxyl groups demonstrated superior cytotoxic effects on several cancer cell lines, particularly, on AML (HL60) cells [45]. In addition, among twenty-one tested derivatives of resveratrol, the compounds with para-hydroxyl groups were shown to be the strongest tyrosinase inhibitors [46]. The importance of α, β-unsaturated carbonyl groups in the anticancer effects of different agents has been explored in several studies. For example, following testing of twenty-seven known electrophilic and antioxidant compounds with drug-like properties it was found that α, β-unsaturated carbonyl-containing agents were selectively cytotoxic to chronic lymphocytic leukemia cells, and that the loss of the α, β unsaturation abrogated this activity [47]. Also, the double bond of the α, β-unsaturated carbonyl moiety of 15-keto prostaglandin E2 was found to be crucial for the inhibition of both STAT3 activation in breast cancer cells and breast tumor growth in a xenograft mouse model [48]. Methyl esterification of the carboxyl group in the phenolic amide javamide-I resulted in enhanced p53-dependent induction of cell death in THP-1 human AML cells [49]. In addition, methyl esterification of the melanin pigment 5,6-dihydroxyindole-2-carboxylic acid was shown to improve its antioxidant activity and photostability [50].

The fact that, when combined with CA, the same chemical structure (KS-3) most effectively promoted both apoptotic cell death (Figure 2f and Figure 4) and elevation of cytosolic Ca^2+^ levels (Figure 3) further supports our previous findings that intracellular calcium mobilization by the KS-3+CA combination is essential for apoptosis induction in AML cells [35]. Studies of the molecular mechanism of HCAD/CA-induced disruption of the intracellular calcium homeostasis are in progress in this laboratory. 

In silico analysis of the chemical interaction between the selected HCADs and CA by means of the molecular quantum mechanical modeling suggests that HCAD molecules can potentially form stable complexes with the CA anion. The binding energy varies between −14 and −19 kcal/mol (Table 1) and is 3–4 times stronger than hydrogen bonds in water. Unique geometry of these complexes implies that the HCAD—CA species, particularly KS-3—CA anion may bind, perhaps selectively, to certain cellular structures, e.g., regulatory proteins involved in the intracellular calcium homeostasis. The lower negative BE, i.e., lower stability, of the KS-6—CA complex compared to that of KS-3—CA correlates with a lower cytotoxic activity of the KS-6+CA combination. However, similar low BE values were also obtained for the non-active KS-1—CA or KS-9—CA complexes. This suggests that chemical stability of putative HCAD—CA species might play a lesser role in their biological activity than, e.g., the specific structures/conformations of the complexes.

Another important finding of this study is that KS-3 and KS-6 also exhibited superior abilities to potentiate the differentiation-inducing effect of calcitriol, applied at a low nanomolar concentration, as compared to the other HCADs, pCouA and FerA (Figure 8). These results indicate that the presence of the three key structural moieties in an HCAD molecule (the para position of the hydroxyl group on the phenolic ring, C7–C8 double bond and methyl ester modification of the carboxyl group) is essential. not only for the cooperation with CA to induce cell death, but, also, for its differentiation-enhancing capacity, although a major requirement for such potentiation appears to be the existence of the double bond (Figure 8 and Suppl. Table S2).

We have recently demonstrated that the transcription factor Nrf2 mediates the enhancing effect of CA on calcitriol-induced differentiation of AML cells and that other structurally distinct Nrf2 activators can also potentiate the differentiation-inducing effects of active vitamin D derivatives [28,38]. These effects were associated with upregulation of different Nrf2 target genes (NQO1, TrxR1 and glutamate-cysteine ligase) both at mRNA and protein levels [27,28,38]. By analogy, the ability to KS-3 and KS-6 (which contain the α, β-unsaturated carbonyl), but not MHP and MHMP (which lack this moiety), to upregulate NQO1 and TrxR1 protein levels (Figure 9) implies the involvement of the Nrf2 signaling pathway in the differentiation-enhancing effects of HCADs containing the C7–C8 carbon double bond. Phenolic acid derivatives, such as caffeic acid phenethyl ester and ethyl ferulate, were also found to activate Nrf2 [51]. Furthermore, it was shown that ethyl esters of ferulic and caffeic acid were capable of inducing differentiation of HL60 cells and enhancing the maturation stimulated by calcitriol [52]. As the presence of α, β-unsaturated carbonyl moiety in various electrophilic compounds is crucial for their activation of the Nrf2/antioxidant response element signaling pathway [47,53,54,55], our present findings further support, though indirectly, the notion that Nrf2 activation by electrophilic phenolic compounds has a key role in the enhancement of calcitriol-induced differentiation of AML cells. 

## 5. Conclusions

In conclusion, the findings presented here revealed the previously unknown chemical nature of the synergy between phenolic compounds in killing AML cells. Particularly, the structure–activity relationship analysis of HCADs resulted in the identification of key structural moieties of HCAD molecules that are critical for the synergy with CA in killing AML cells. Notably, the same structural features of HCADs were found to be important for the synergistic cooperation of HCADs and the active vitamin D metabolite calcitriol in inducing the enhanced differentiation of AML cells. The above findings may represent a novel approach for the rational design of synergistically acting AML drugs based on the prototype hydroxycinnamate-phenolic diterpene and hydroxycinnamate-calcitriol combinations tested in this study.

## Figures and Tables

**Table 1:** Binding energy of selected carnosic acid anion-hydroxycinnamic acid derivative molecular complexes.

Molecular Complex ^a^	Binding Energy [kcal/mol] ^b^
Carnosic acid anion—KS-1	−17
Carnosic acid anion—KS-3	−19
Carnosic acid anion—KS-6	−16
Carnosic acid anion—KS-7	−14
Carnosic acid anion—KS-9	−16

^a^ The full names of KS-1, KS-3, KS-6, KS-7 and KS-9 compounds are defined in the Experimental section. ^b^ Binding energies were calculated as described in the Experimental Section.

## Data Availability

Data is contained within the article or Supplementary Material.

## Supplementary Materials

The following are available online at https://www.mdpi.com/article/10.3390/biomedicines9111517/s1, Figure S1: Comparison between the apoptosis-inducing effects of the combinations of KS-3 and KS-6 with carnosic acid, Figure S2: The additional 3-hydroxyl group on the phenolic ring of KS-3 (as in methyl caffeate) abolishes its ability to cooperate with carnosic acid, Table S1: The Cartesian coordinates of the carnosic acid anion—KS-x molecular complexes, Table S2: KS-3 and KS-6 enhance calcitriol-induced differentiation of AML cells significantly stronger than their chemical analogs.
