# Supplementary material for: Structure-Activity Relationship of Hydroxycinnamic Acid Derivatives for Cooperating with Carnosic Acid and Calcitriol in Acute Myeloid Leukemia Cells
Source: Biomedicines. 2021 Oct 21;9(11):1517. doi: 10.3390/biomedicines9111517 (PMC8615284; doi:10.3390/biomedicines9111517)
Supplement: Supplementary file 1 [file biomedicines-09-01517-s001.zip › biomedicines-1320881-supplementary.pdf]

Supplementary Material

Structure-activity relationship of hydroxycinnamic acid derivatives for cooperating with carnosic acid and calcitriol in acute myeloid leukemia cells

Aviram Trachtenberg [1], Katarzyna Sidoryk[2], Somaya Alreate[1], Suchismita Muduli1, Andrzej Leś[3], Marcin Cybulski[2], Michael Danilenko[1]*

[1] Department of Clinical Biochemistry and Pharmacology, Faculty of Health Sciences, Ben-Gurion University of the Negev, Beer Sheva 8410501, Israel; aviramtr@post.bg.ac.il (A.T.), alreates@post.bgu.ac.il (S.A.), muduli@post.bgu.ac.il (S.M.), misha@bgu.ac.il (M.D.)
[2] Department of Pharmacy, Cosmetic Chemicals and Biotechnology, Team of Chemistry, Łukasiewicz Research Network - Industrial Chemistry Institute, Warsaw 01-793, Poland; sidorykk@gmail.com (K.S.), m.cybulski@ifarm.eu (M.C.)
[3] Faculty of Chemistry, University of Warsaw, Warsaw 02-093, Poland; ales@tiger.chem.uw.edu.pl (A.L.)
* Correspondence: misha@bgu.ac.il; Tel.: +972-8-647-9979

1. Supplementary methods

1.1. Acridine orange and ethidium bromide staining

Cells (5×10^4/ml) were collected by centrifugation and double stained with 14 μg/ml acridine orange and 14 μg/ml ethidium bromide, as described [1]. Nuclear morphology of stained cells was examined by fluorescent microscopy (Olympus BX60, Olympus corporation, Tokyo, Japan) at a magnification of 400x. The percentage of apoptotic cells was calculated following manual counting of cells with shrunk green (early apoptosis) or red nuclei (late apoptosis) and this number was divided by the total number of cells in an image. Nuclei stained red with no signs of chromatin shrinkage were considered necrotic and were not counted for apoptosis. A minimum of 100 cells per sample were counted.

1.2. Assessment of cell viability using the CellTiter-Glo® ATP assay

Cell viability was determined on the basis of changes in cellular ATP levels using the CellTiter-Glo Luminescent Cell Viability Assay kit (Promega, Madison, WI) according to the manufacturer's instructions. Briefly, KG-1a cells were incubated with test agents for 72 h and then 100 μl cell suspension was transferred into a white-bottom 96-well plate followed by the addition of 100 μl ATP assay buffer. The plate was shaken for 2 min and kept at room temperature for 10 min. Luminescence was then measured in quadruplicate using a SpectraMax Paradigm (Molecular Devices Co., Sunnyvale, CA, USA) plate reader. A similar procedure was used for the preparation of plate blank wells, except that complete cell growth medium was added instead of cell suspension.

1.3. Combination index analysis

The interaction between MCAF and CA in inducing cytotoxicity was assessed by the combination index (CI) analysis using Calcusyn 2.0 software (BIOSOFT, United Kingdom). The CI values were calculated based on the extent of reduction in cell viability (fraction affected) by each agent individually and by the combination at a constant ratio. CI values of <1, 1, and >1 show synergism, additivity, and antagonism, respectively.

2. Supplementary figures

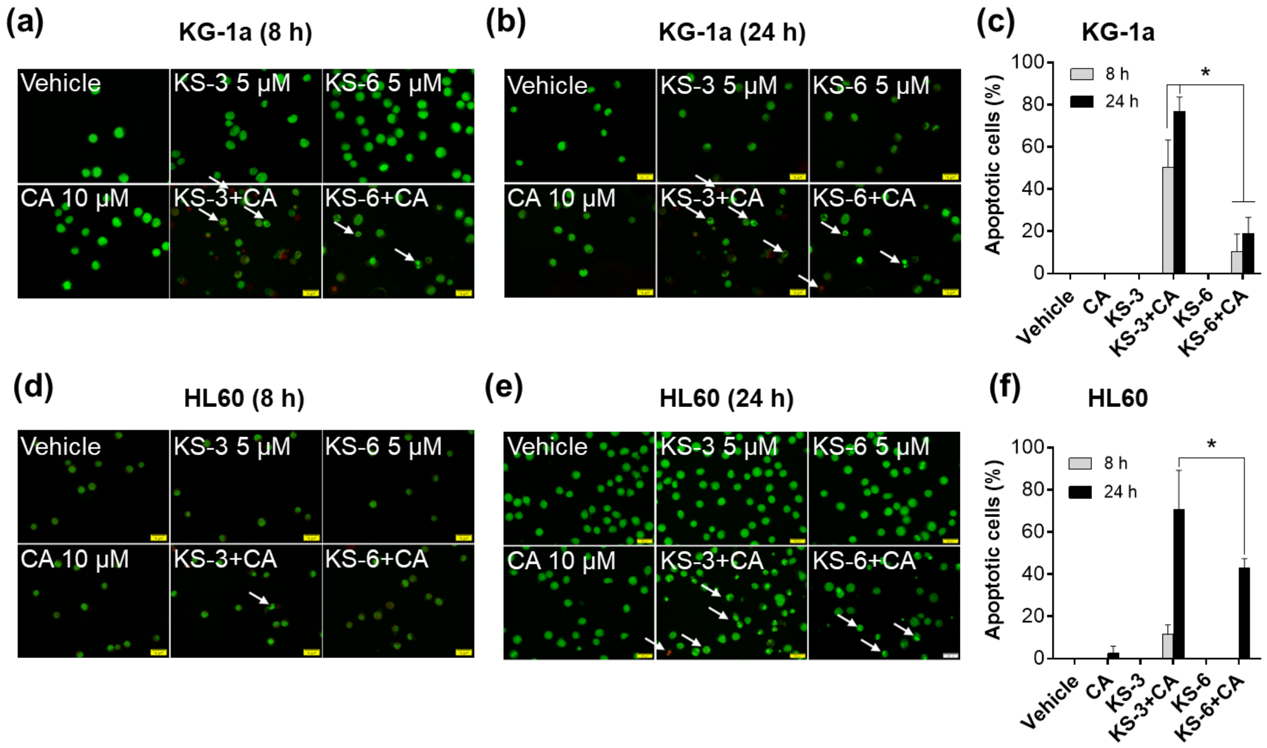

Figure S1. Comparison between the apoptosis-inducing effects of the combinations of KS-3 and KS-6 with carnosic acid. KG-1a cells were cultured with KS-3, KS-6, carnosic acid (CA), or their combinations, for 8 or 24 h. (**a**, **b**, **d**, **e**) Changes in the nuclear morphology of KG-1a (**a**, **b**) and HL60 (**d**, **e**) cells stained with acridine orange (green) and ethidium bromide (red) were examined by fluorescence microscopy. Arrows indicate late apoptotic cells and/or apoptotic bodies. (**c**, **f**) Averaged percentages of apoptotic cells in the KG-1a (**c**) and HL60 (**f**) cell cultures. Cells were enumerated manually (≥100 cell counts per sample). The data are the means ± SD (n = 3). *, $p < 0.01$; significant differences between the indicated groups; Student's t test.

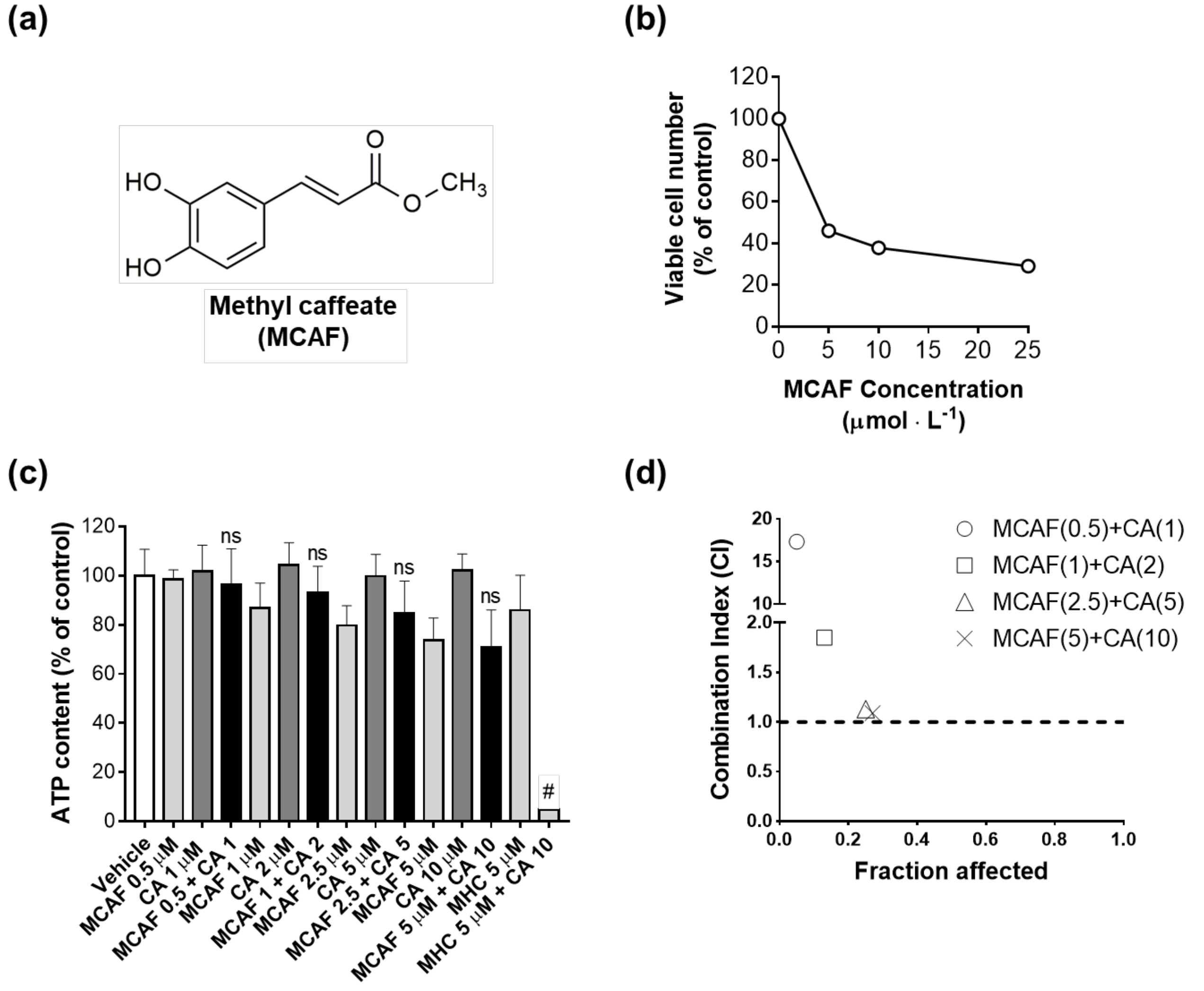

Figure S2. The additional 3-hydroxyl group on the phenolic ring of KS-3 (as in methyl caffeate) abolishes its ability to cooperate with carnosic acid. (**a**) Molecular structure of methyl caffeate (MCAF). (**b-d**) KG-1a cells were treated with the indicated compounds, for 72 h followed by the Trypan Blue exclusion assay (**b**) or the ATP-based cell viability assay (**c, d**). (**b**) Concentration-dependent effect of MCAF alone on the number of viable cells. (**c**) The effects of MCAF and CA, alone and in combination, at the indicated concentrations on cell viability. The data are the means ± SD (n=12-16). #, $p < 0.05$, effect of the MHC+CA combination *vs*. sum of the effects of single agents; Student's t test, ns – non-significant. (**d**) Combination index (CI) analysis of cytotoxicity for the indicated MCAF+CA combinations (μM). The CI values are plotted against the levels of the fraction affected.

3. Supplementary tables

Table S1. The Cartesian coordinates of the carnosic acid anion --- KS-x molecular complexes.

x = 1, 3, 6, 7, 9

Carnosic acid anion --- KS-1

74

O	2.1072784098	-1.5017772688	4.2508048199
O	3.4353899221	-2.4632101524	2.7615798166
C	2.3238292147	0.3081460509	-0.3454162704
C	2.0906780966	1.6975404436	-0.212635129
C	2.3372213208	-0.2113394466	-1.6534111981
C	1.8713806635	2.497998054	-1.335979775
C	2.1112440113	0.5793952601	-2.7757926533
C	2.5496063785	-0.6206404965	0.7589133628
C	1.8711913308	1.9431980032	-2.6152700465
C	2.3871048522	-0.4292880546	2.0821982852
C	2.6808465625	-1.5436086339	3.0123670347
C	1.0860563913	-0.5354067559	4.5489061487
O	-4.446721522	0.8369577194	0.0685515751
O	-3.4348738867	-0.0520367993	1.8273010344
O	-1.1438789882	-0.9372850459	2.2197716688
O	0.2582856702	-3.0912543898	1.866792628
C	-2.0009115688	1.7157332968	-1.2305138687
C	-1.9882025291	0.8962923654	0.0982133802
C	-2.5942000174	3.1724919974	-1.1740142974
C	-1.1951263921	1.721046511	1.1836465897
C	-2.4689424752	3.7128257299	0.2703547987
C	-2.513674531	0.8667293634	-2.3999386016
C	-1.1705817049	3.238290886	0.925134423
C	-1.2880168077	-0.4629474839	-0.1574900524
C	-1.4992512862	-0.2363653246	-2.6956170762
C	-1.742580477	4.0502112837	-2.1206306114
C	-4.0607763074	3.2980822552	-1.6283592532
C	-1.0413785182	-0.9696387218	-1.4506055399
C	-3.4072740968	0.5644323459	0.6541673815
C	-0.8765101204	-1.2357151647	0.9565831711
C	-0.3214998017	-2.1587992257	-1.6185957929
C	0.1709919748	-2.8987608758	-0.5329402445
C	-0.1342793899	-2.4234371984	0.7377125316
C	0.9616914248	-4.1926188002	-0.659506547
C	1.6054596564	-4.399211835	-2.0362043237
C	0.0810217325	-5.4028850711	-0.28913713
H	2.4947144646	-1.2772126923	-1.7713309245
H	2.8703125288	-1.6154217531	0.4577842505
H	1.6986981054	3.5628374679	-1.1996227244
H	2.1049703851	0.1301292259	-3.7636735207
H	2.0137238589	0.5075161446	2.4657791371
H	1.5166046999	0.4648205549	4.6894986037
H	0.3114411777	-0.5101169568	3.7742645294
H	0.6490932081	-0.8603975577	5.496192424
H	-0.9284774397	1.8623412587	-1.4239647204
H	-0.1783263738	1.3364928362	1.2503960891

H	-1.6391935419	1.5434585682	2.1622900914
H	-3.3173787656	3.3513097614	0.8642102524
H	-2.535326561	4.8095560134	0.2594114193
H	-2.6477756097	1.4839767667	-3.296661832
H	-3.4913614225	0.4511623437	-2.1420374438
H	-0.9965450818	3.7661211091	1.8719921867
H	-0.3313343728	3.5040871303	0.2660058306
H	-0.6226353221	0.2166935357	-3.1837721942
H	-1.910184581	-0.957338288	-3.415060828
H	-0.700462357	4.1202627481	-1.7905872227
H	-1.7340824961	3.6340369293	-3.1344439253
H	-2.1485328667	5.0674960546	-2.1806659054
H	-4.1752849345	3.0519718986	-2.6894988051
H	-4.7014040626	2.6338968606	-1.0516505208
H	-4.3957405696	4.3358465263	-1.497363695
H	-0.1327933983	-2.5016858597	-2.6323700301
H	1.7664285344	-4.1435652349	0.0869976023
H	-2.4840212518	-0.3978879538	2.10674539
H	-0.1675002576	-2.5587044549	2.5730278735
H	0.847235739	-4.5351935023	-2.8161746285
H	2.2362103383	-5.2954532274	-2.0315701309
H	2.2300770215	-3.547391845	-2.326287601
H	-0.3252026522	-5.2783173101	0.7171043107
H	0.6581800884	-6.3361146378	-0.3188651553
H	-0.7552917472	-5.4946858453	-0.992529143
O	2.099213929	2.24767341	1.0419530993
H	1.6790483672	2.5783884542	-3.4748821529
H	1.6544600797	3.104926821	1.0081300185

Carnosic acid anion --- KS-3

74

O	2.0540265393	-1.6906974864	4.2134697326
O	3.2999071427	-2.7594930298	2.7311377512
C	2.3783538147	0.0344513627	-0.3994737861
C	2.3058858	1.423185243	-0.1938721651
C	2.2266614532	-0.4407120085	-1.7154618418
C	2.0400667789	2.2949350269	-1.2431964093
C	1.9546377244	0.4205713261	-2.7711881882
C	2.5908111421	-0.9175919867	0.6873277227
C	1.8279620009	1.7913941933	-2.5330374568
C	2.3162931565	-0.7198596543	1.9902296575
C	2.5904879416	-1.7982043789	2.9655928755
C	1.1136352689	-0.649236115	4.526169163
O	-4.3090810858	1.2074499517	0.2037555514
O	-3.3532496059	0.2169009288	1.9393891326
O	-1.1692648836	-0.9182607116	2.2872363447
O	-0.0508708742	-3.2288373116	1.9268973825
C	-1.8285645581	1.7814741745	-1.1827050511
C	-1.8622340142	0.9811247758	0.1564141655
C	-2.2806310606	3.2887971164	-1.1285454319
C	-0.9470509436	1.7335742695	1.1911862588
C	-2.1472852438	3.8108092946	0.3204378331
C	-2.4549869503	0.9780553839	-2.3282059658
C	-0.8910471672	3.2642472711	1.000761792
C	-1.3195243777	-0.4504854943	-0.0936634962
C	-1.5770936504	-0.2341334073	-2.6292937702
C	-1.3270391944	4.0868130126	-2.0505400954
C	-3.7173906573	3.5515373806	-1.6204672485
C	-1.1695719433	-0.9983440219	-1.3856450745
C	-3.2908413295	0.8172999634	0.7601303152
C	-0.9681520019	-1.2547080086	1.0186497807
C	-0.6115432971	-2.2710601125	-1.5549484747
C	-0.1958956134	-3.0617513054	-0.4720478624
C	-0.3862485603	-2.5285081691	0.7982956248
C	0.4028298998	-4.455188497	-0.6014741671
C	1.9032914918	-4.4593820102	-0.2469567417
C	0.1722896887	-5.1026273622	-1.9726389849
H	2.2648097852	-1.5094225071	-1.8957505754
H	2.9805656014	-1.8930374941	0.4106141718
H	1.9866514304	3.3656899427	-1.0647763865
H	1.7936733408	0.0466781244	-3.7763895294
H	1.8299082218	0.1900976277	2.3175009129
H	1.6143324934	0.3253584019	4.5988570758
H	0.298583122	-0.6070229462	3.7946643254
H	0.7157726991	-0.9075263917	5.5101752757
H	-0.7519808768	1.8198375751	-1.4004602463
H	0.0585118813	1.3179488508	1.1067159559
H	-1.2809271166	1.5020411163	2.2001932723
H	-3.0322524903	3.5037267603	0.8891777884
H	-2.1431371528	4.9100200371	0.3137940361
H	-2.537945924	1.5939119721	-3.2319591473
H	-3.4671450272	0.6777569622	-2.0439812894
H	-0.7451070113	3.7488564317	1.9743015414
H	-0.0126069973	3.532633738	0.4001839242

H	-0.6729813925	0.1158396522	-3.1491213213
H	-2.0842799543	-0.9139850183	-3.3268232747
H	-0.3068461134	4.1031547852	-1.6484455963
H	-1.2903296313	3.6394490741	-3.0510641702
H	-1.6594279891	5.1263952833	-2.1566824399
H	-3.8231511329	3.346112491	-2.6910389374
H	-4.4260600193	2.9307577967	-1.0753443104
H	-3.9676068679	4.6096866456	-1.4666605953
H	-0.5143344744	-2.6530117185	-2.5675097824
H	-0.0971771151	-5.0782200241	0.1532527439
H	-2.441139571	-0.2353548716	2.1970303142
H	-0.4017350538	-2.6455488102	2.6344492147
H	2.4689932842	-3.8601554536	-0.9722916275
H	2.3082341156	-5.4791972902	-0.2754484936
H	2.0757165576	-4.0516313603	0.7517601602
H	-0.8884540129	-5.1058766843	-2.2439961555
H	0.5312390404	-6.1382588932	-1.9710761639
H	0.7169713574	-4.5694231437	-2.7612316531
O	1.5043136677	2.5977833303	-3.5921176861
H	1.1762686171	3.4399835317	-3.2495392877
H	2.4653227605	1.8270110805	0.8008279505

Carnosic acid anion --- KS-6

78

O	-1.977716	3.505165	2.060545
O	-3.652297	1.974377	2.033757
C	-0.420803	-0.965762	2.246933
C	0.847137	-0.684588	2.805049
C	-0.571961	-2.134718	1.488632
C	1.922039	-1.52001	2.553811
C	0.508876	-2.981004	1.244888
C	-1.518185	-0.001126	2.280408
C	1.764366	-2.672372	1.75303
C	-1.394605	1.314201	2.543226
C	-2.476745	2.24758	2.205409
C	-2.82866	4.395492	1.328974
O	2.837262	-3.469761	1.480898
H	-1.521358	-2.335719	1.007085
H	-2.484583	-0.346598	1.921295
H	0.401946	-3.857885	0.61611
H	-0.44134	1.768741	2.788611
H	-2.950325	4.011778	0.311724
H	-3.808228	4.491622	1.807044
H	-2.312925	5.357394	1.31518
H	0.98287	0.212421	3.396074
O	3.216069	-1.331462	2.99796
C	3.484353	-0.197705	3.8086
H	4.548181	-0.24079	4.049076
H	2.898338	-0.225502	4.73693
H	3.271063	0.73594	3.275263
H	3.61488	-3.027747	1.854554
O	1.551024	2.026298	-3.413802
O	-0.198887	2.847853	-2.327314
O	-1.777882	1.935482	-0.650325
O	-3.951764	0.432097	-0.472175
C	2.136148	-0.030174	-1.44336
C	0.900714	0.916048	-1.32049
C	3.562526	0.630479	-1.34066
C	1.008276	1.64281	0.065799
C	3.446661	1.991752	-0.617995
C	1.980374	-1.004738	-2.614756
C	2.451435	1.921204	0.538891
C	-0.401853	0.068467	-1.375157
C	0.84121	-1.969519	-2.303733
C	4.447728	-0.327091	-0.507671
C	4.266657	0.853961	-2.693521
C	-0.413217	-1.270774	-1.817853
C	0.789331	1.977171	-2.455256
C	-1.632803	0.661268	-0.971739
C	-1.594522	-2.020711	-1.786111
C	-2.804908	-1.487016	-1.321972
C	-2.796225	-0.154362	-0.916601
C	-4.108119	-2.269365	-1.238166
C	-4.472384	-2.572962	0.22949
C	-4.116564	-3.556537	-2.071814

H	2.047867	-0.643704	-0.533418
H	0.514773	1.006751	0.794655
H	0.434526	2.566089	0.045435
H	3.117185	2.749273	-1.337998
H	4.440036	2.302717	-0.264118
H	2.903607	-1.575426	-2.77086
H	1.786041	-0.438304	-3.529898
H	2.474066	2.850542	1.12249
H	2.769927	1.125381	1.223869
H	1.185529	-2.669867	-1.526509
H	0.600894	-2.584081	-3.181702
H	4.080752	-0.448588	0.517712
H	4.46914	-1.324755	-0.962082
H	5.480915	0.03947	-0.457861
H	4.514919	-0.093296	-3.184074
H	3.633591	1.436821	-3.360606
H	5.210962	1.390002	-2.528527
H	-1.556126	-3.049048	-2.134975
H	-4.89718	-1.610739	-1.626623
H	-0.882865	2.560421	-1.571543
H	-3.677181	1.333489	-0.212817
H	-3.726199	-3.238305	0.681937
H	-5.448918	-3.069683	0.300338
H	-4.514868	-1.646979	0.808001
H	-3.848051	-3.360866	-3.115076
H	-5.110431	-4.01878	-2.052228
H	-3.40555	-4.291966	-1.676557

Carnosic acid anion --- KS-7

```
73

 O   2.2500225356  -1.8472249593  3.6724389848
 C   2.4311686389  0.4032454856  -0.0118267958
 C   2.2092470442  1.7876557364  -0.2150412856
 C   2.5691979436  -0.3949592329  -1.163154949
 C   2.0749883017  2.309438852  -1.5029576625
 C   2.4438564362  0.1208718541  -2.4483842464
 C   2.4042528186  -0.2732108193  1.2825395147
 C   2.1805791857  1.4795466868  -2.6189539163
 C   2.0307296822  0.2114304369  2.48505315
 C   1.807855151  -0.7029349509  3.6258607889
 C   0.9172266047  -0.1590985382  4.7278302651
 O   -4.3620550325  0.4847784407  0.2559585894
 O   -3.1175128727  -0.2050723964  1.9857919699
 O   -0.8614076817  -1.1288177245  2.0907895987
 O   0.6532907268  -3.2317645711  1.474563282
 C   -2.1014627514  1.6367049689  -1.1719778613
 C   -1.926759905  0.7722609501  0.1164056671
 C   -2.7800056465  3.0440060605  -0.991094495
 C   -1.0574514149  1.5984008655  1.132202169
 C   -2.5268554729  3.5411769327  0.4526183216
 C   -2.6654908203  0.807273687  -2.329723841
 C   -1.1383274926  3.1224124709  0.9362697073
 C   -1.1978778021  -0.5435204806  -0.252674665
 C   -1.6108944756  -0.2102517388  -2.7553984522
 C   -2.1036517406  4.0150874814  -1.9862242284
 C   -4.2940543009  3.0710127232  -1.2702009105
 C   -1.0322300501  -0.9774969483  -1.5832481896
 C   -3.2728850777  0.3310067531  0.8246656909
 C   -0.6719335105  -1.3650385778  0.7732028236
 C   -0.2893152683  -2.130169776  -1.863639509
 C   0.3144323661  -2.897446561  -0.8650047924
 C   0.1008822849  -2.4979604871  0.4562856359
 C   1.1497119077  -4.1399831948  -1.1324390195
 C   1.7143564305  -4.2116752519  -2.557159343
 C   0.3514243184  -5.4153641406  -0.7973168577
 H   2.7132342215  -1.4611137766  -1.0272216099
 H   2.599464135  -1.3438102751  1.2454826655
 H   1.8819866559  3.3730812781  -1.6229300386
 H   2.5152168021  -0.5401418235  -3.3059711783
 H   1.7513644398  1.2481510204  2.6121086359
 H   1.2215518852  0.8481123203  5.0339483417
 H   -0.097824811  -0.0943149179  4.3202023622
 H   0.92633032  -0.8357232124  5.5842097075
 H   -1.0617991816  1.866774998  -1.4570746043
 H   -0.0209449542  1.2759788962  1.0693855171
 H   -1.3904554791  1.3625428259  2.1421922599
 H   -3.2790225969  3.1047993314  1.1207560216
 H   -2.6580239035  4.6315284869  0.4949665196
 H   -2.9197281836  1.4468476753  -3.1840784116
 H   -3.5828014576  0.315761477  -1.9959052592
 H   -0.8790306243  3.6265809021  1.8762535983
 H   -0.3963772948  3.4576819861  0.1941733058
 H   -0.7943467386  0.3192701185  -3.2729773334
```

H	-2.0238312629	-0.9195316062	-3.485347549
H	-1.0373528626	4.151208795	-1.7695481548
H	-2.1816893218	3.6395256691	-3.0128464526
H	-2.5827428203	5.0015332265	-1.955049576
H	-4.5120192682	2.8618489874	-2.3234293524
H	-4.8028817191	2.3259716169	-0.6599467692
H	-4.6896907858	4.0715309246	-1.0478923736
H	-0.167188428	-2.4143588049	-2.9053615819
H	1.9987712355	-4.1054248962	-0.4358644571
H	-1.860263126	-0.698837853	2.1598022346
H	0.4294745579	-2.7627011308	2.2970849709
H	0.9175425781	-4.3437344928	-3.297987587
H	2.3967310656	-5.0637197152	-2.6553150311
H	2.2634536278	-3.3007165574	-2.8196850784
H	0.0064763698	-5.3820087535	0.2386601009
H	0.96604476	-6.3149143497	-0.9317055622
H	-0.5245611195	-5.4981762197	-1.4513709481
O	2.1283814228	2.6077852961	0.8799985176
H	2.0549448563	1.8985033759	-3.6130795923
H	1.6126954589	3.3911174132	0.6464629261

Carnosic acid anion --- KS-9

73

O	3.101960857	-2.6224915071	2.7999767422
C	2.4606675353	0.144197734	-0.4553404354
C	2.3200928694	1.5290461986	-0.2621282369
C	2.3624109595	-0.3477628486	-1.7698675786
C	2.0342436377	2.379601027	-1.3231369646
C	2.0653417588	0.4911659493	-2.836756498
C	2.6475734038	-0.7855769152	0.6553067764
C	1.8650144041	1.8564789375	-2.6107604209
C	2.2109517979	-0.6014961931	1.9153126211
C	2.3635989507	-1.6536581276	2.9450788634
C	1.5410862751	-1.449815543	4.2041476728
O	-4.3278121408	1.234835722	0.1245301608
O	-3.3613987002	0.4318716676	1.973991863
O	-1.3428379887	-0.8416829136	2.3512181682
O	-0.2735244869	-3.1964836982	2.0572050386
C	-1.8161789362	1.8257483709	-1.2002753236
C	-1.8809103257	1.0563317212	0.1550518009
C	-2.2871889864	3.3270313362	-1.1773389713
C	-0.9516360739	1.8158177386	1.1643494753
C	-2.181439545	3.8692805502	0.2668295772
C	-2.4072685201	0.9954431962	-2.3438016319
C	-0.9286892864	3.3501032858	0.9761540568
C	-1.3565825748	-0.3856177715	-0.0504538713
C	-1.502954415	-0.2080511729	-2.5973461673
C	-1.3338456748	4.1256182224	-2.098928462
C	-3.7218531541	3.5585195101	-1.6896618051
C	-1.1414862346	-0.9500224842	-1.3259541854
C	-3.3265271495	0.9138441075	0.7751683701
C	-1.0801539113	-1.1976081128	1.0714893124
C	-0.5814568396	-2.2273919835	-1.4497057045
C	-0.244193625	-3.0164954257	-0.3438058711
C	-0.5196610496	-2.477438563	0.9116652801
C	0.3664584819	-4.4062966598	-0.4330729872
C	1.863797292	-4.3771631512	-0.0674142349
C	0.1564820883	-5.0876494002	-1.7913555577
O	1.5111037043	2.6367065247	-3.6782070393
H	2.4573299059	-1.4153713661	-1.9405375462
H	3.1191702542	-1.7404878158	0.4329938699
H	1.9228006653	3.446851587	-1.1531882648
H	1.9368464958	0.1032345925	-3.8413863687
H	1.6213723051	0.2710601938	2.1810173571
H	1.968655648	-0.6253257357	4.79028197941
H	0.5176730599	-1.1641707271	3.9390010684
H	1.552479687	-2.3607357082	4.8045406131
H	-0.7349262824	1.8755330101	-1.3980641458
H	0.0619100968	1.4254794938	1.0474162215
H	-1.2621696049	1.5756570724	2.1786164847
H	-3.0706845771	3.5561079989	0.8242859415
H	-2.1912274154	4.9685701666	0.2467752699
H	-2.4798667552	1.5903193944	-3.2626583586
H	-3.4186605052	0.6857684089	-2.0673652272
H	-0.8177133781	3.8396347147	1.9517715656
H	-0.042947688	3.6410783105	0.3956391468

H	-0.5828579922	0.1420780326	-3.0881256432
H	-1.9756721491	-0.9064141617	-3.3009808854
H	-0.3179102631	4.1621371589	-1.6869017641
H	-1.283302647	3.6652782092	-3.0935316295
H	-1.6788156176	5.1591141733	-2.2235931414
H	-3.8104993686	3.3369000063	-2.7589657882
H	-4.419198131	2.9265633889	-1.1415701151
H	-3.9933846233	4.6140123034	-1.5527074303
H	-0.4256985014	-2.6174123241	-2.4520065609
H	-0.1335013934	-5.016720183	0.3311147254
H	-0.7031331161	-2.6543149214	2.7470633531
H	2.4225435218	-3.7850737734	-0.8035904974
H	2.2868134304	-5.390045841	-0.0659203126
H	2.023807212	-3.9378164364	0.9197813643
H	-0.9005409701	-5.1006080009	-2.076273546
H	0.5185858176	-6.1216656921	-1.7594032749
H	0.7103195502	-4.5731769506	-2.5857339616
H	1.115802462	3.4537820654	-3.3447066653
H	2.4359253045	1.9442680586	0.7336834454
H	-2.2801674834	-0.2233325232	2.286679747

Table S2. KS-3 and KS-6 enhance calcitriol-induced differentiation of AML cells significantly stronger than their chemical analogs.

(a)

Dunnett's multiple comparisons test	Mean Diff.	95.00% CI of diff.	Adjusted P Value	Mean Diff.	95.00% CI of diff.	Adjusted P Value
		**HL60**			**U937**	
KS-3+1,25D vs. KS-1+1,25D	18.31	4.478 to 32.15	**0.0037**	21.61	12.5 to 30.73	**0.0001**
KS-3+1,25D vs. KS-2+1,25D	17.44	3.118 to 31.76	**0.0092**	19.66	9.676 to 29.65	**0.0001**
KS-3+1,25D vs. KS-6+1,25D	2.291	-11.54 to 16.13	**0.9993**	6.321	-3.664 to 16.31	**0.4241**
KS-3+1,25D vs. KS-7+1,25D	22.46	7.513 to 37.4	**0.0007**	16.74	7.912 to 25.56	**0.0001**
KS-3+1,25D vs. KS-8+1,25D	25.61	11.29 to 39.94	**0.0001**	23.34	14.74 to 31.93	**0.0001**
KS-3+1,25D vs. KS-9+1,25D	18.69	4.365 to 33.01	**0.0043**	21.52	13.11 to 29.92	**0.0001**
KS-3+1,25D vs. KS-12+1,25D	26.63	11.69 to 41.58	**0.0001**	22.13	13.01 to 31.24	**0.0001**
KS-3+1,25D vs. pCouA+1,25D	27.23	13.39 to 41.06	**0.0001**	21.01	12.41 to 29.6	**0.0001**
KS-3+1,25D vs. FerA+1,25D	30.99	16.04 to 45.93	**0.0001**	17.02	6.334 to 27.71	**0.0003**
KS-3+1,25D vs. MHP+1,25D	43.54	28.59 to 58.48	**0.0001**	30.86	21.37 to 40.35	**0.0001**
KS-3+1,25D vs. MHMP+1,25D	43.99	29.04 to 58.93	**0.0001**	32	22.51 to 41.49	**0.0001**

(b)

Dunnett's multiple comparisons test	Mean Diff.	95.00% CI of diff.	Adjusted P Value	Mean Diff.	95.00% CI of diff.	Adjusted P Value
		**HL60**			**U937**	
KS-6+1,25D vs. KS-1+1,25D	16.02	2.187 to 29.86	**0.015**	**15.29**	**5.308 to 25.28**	**0.0005**
KS-6+1,25D vs. KS-2+1,25D	15.15	0.8264 to 29.47	**0.0328**	**13.34**	**2.555 to 24.13**	**0.0078**
KS-6+1,25D vs. KS-3+1,25D	2.291-	-16.13 to 11.54	**0.9993**	**6.321-**	**-16.31 to 3.664**	**0.4241**
KS-6+1,25D vs. KS-7+1,25D	20.17	5.222 to 35.11	**0.0029**	**10.42**	**0.6952 to 20.14**	**0.0296**
KS-6+1,25D vs. KS-8+1,25D	23.32	9.002 to 37.64	**0.0002**	**17.01**	**7.502 to 26.53**	**0.0001**
KS-6+1,25D vs. KS-9+1,25D	16.39	2.074 to 30.72	**0.0167**	**15.2**	**5.856 to 24.54**	**0.0002**
KS-6+1,25D vs. KS-12+1,25D	24.34	9.397 to 39.28	**0.0002**	**15.8**	**5.82 to 25.79**	**0.0003**
KS-6+1,25D vs. pCouA+1,25D	24.94	11.1 to 38.77	**0.0001**	**14.68**	**5.172 to 24.2**	**0.0005**
KS-6+1,25D vs. FerA+1,25D	28.7	13.75 to 43.64	**0.0001**	**10.7**	**-0.7377 to 22.14**	**0.0781**
KS-6+1,25D vs. MHP+1,25D	41.25	26.3 to 56.19	**0.0001**	**24.54**	**14.21 to 34.86**	**0.0001**
KS-6+1,25D vs. MHMP+1,25D	41.69	26.75 to 56.64	**0.0001**	**25.68**	**15.35 to 36**	**0.0001**

Effects of the KS-3+1,25D (**a**) and KS-6+1,25D (**b**) combinations were compared to those of the combinations of *p*-coumaric acid (pCouA), ferulic acid (FerA) or their other tested derivatives and 1,25D. The percentages of HL60 and U937 cells double-positive for CD14 and CD11b were obtained from the data shown in **Figure 8**. The differences between various treatment groups were analyzed by one-way ANOVA with Dunnett's post-hoc test (n=5). The differences between means (Mean Diff), 95% confidence intervals for the Mean Diff (95.00% CI of diff.) and P values (Adjusted P Value) are presented.

Reference

1. Pesakhov, S.; Khanin, M.; Studzinski, G.P.; Danilenko, M. Distinct combinatorial effects of the plant polyphenols curcumin, carnosic acid, and silibinin on proliferation and apoptosis in acute myeloid leukemia cells. Nutr Cancer 2010, 62, 811-824, doi:10.1080/01635581003693082.
